# Intraoperative CT evaluation of repositioning accuracy in zygomaticomaxillary complex fractures: 1-point versus multiple-point fixation

**DOI:** 10.1186/s13005-025-00558-x

**Published:** 2025-12-13

**Authors:** Andreas Sakkas, Johannes Schulze, Mario Scheurer, Majeed Rana, Robin Kasper, Marcel Ebeling, Alexander Schramm, Frank Wilde, Marc Knitz

**Affiliations:** 1 Department of Oral and Maxillofacial Surgery, University Hospital Ulm, Albert-Einstein-Allee 11, Ulm, 89081 Germany; 2 Department of Oral and Plastic Maxillofacial Surgery, Military Hospital Ulm, Ulm, Germany

**Keywords:** Zygomaticomaxillary fractures, Keen’s approach, 1-point fixation, Osteosynthesis, Surgical accuracy

## Abstract

**Purpose:**

The increasing interest in minimally invasive procedures, which carries minimal risk for wound complications and scarring while also providing predictable outcomes, has brought attention to the concept of 1-point fixation in treating zygomaticomaxillary (ZMC) fractures. The primary aim of this study was to compare the intraoperative accuracy of repositioning following osteosynthesis with 1-point fixation versus multiple-point fixation in unilateral fractures of the ZMC complex. The secondary aim was to evaluate associations between trauma- and procedure-specific factors and the surgical outcomes of both fixation methods.

**Methods:**

In this retrospective cohort single-centre study, patients who underwent surgical treatment for unilateral ZMC fractures using either 1-point or multiple-point fixation over a 7-year period were included. Forty-two patients were treated with 1-point fixation through an intraoral Keen’s approach (group 1), while 20 patients were treated with multiple-point fixation through intraoral and extraoral approach (group 2). Demographic, clinical, radiological, and treatment data were analyzed. The degree of repositioning including protrusion difference of the zygoma (height, width, diagonal), malar difference using an asymmetry index, and difference in the intraorbital volume were measured comparing preoperative and intraoperative CT imaging. Multivariable analyses were conducted to compare the surgical outcomes in both groups and identify associations between the trauma- and procedure-specific variables and the intraoperative accuracy of fracture repositioning.

**Results:**

A total of 62 patients with 62 unilateral ZMC fractures were included in the analysis. Violence was the most common mechanism of injury (*n* = 16; 25.8%). Forty-two patients underwent treatment with 1-point fixation, while 20 patients received multiple-point fixation. The intraoperative malar width difference and malar diagonal difference were significantly higher in group 1 (*p* = 0.003 / *p* = 0.036). No difference was detected in the intraorbital volume between the two fixation groups intraoperatively. Univariate analysis revealed that an interval between trauma-surgery of 3–7 days was significantly associated a better repositioning result in terms of intraorbital volume difference after multiple-point fixation (*p* = 0.03).

**Conclusions:**

This study shows that multiple-point fixation achieves superior accuracy in restoring zygomatic geometry, especially malar projection and transverse symmetry, while both techniques adequately restore intraorbital volume. Single-point fixation remains a viable, less invasive option for non-comminuted ZMC fractures when guided by intraoperative 3D imaging. The lack of revision surgeries in either group supports a tailored, fracture-specific strategy that balances precision with minimal invasiveness, consistent with trends toward individualized, resource-efficient trauma care.

## Introduction

The zygomaticomaxillary complex (ZMC) is a key structural buttress of the face, influencing both function and aesthetics while protecting the orbital contents [1–7]. ZMC fractures are among the most common facial injuries at Level I trauma centres, with etiology and severity varying by sociodemographic, cultural, and environmental factors [1, 8–10].

Clinical decision-making for displaced or multifragmentary ZMC fractures can be challenging. Clinical symptoms such as midfacial asymmetry, diplopia from impaired extraocular muscle function, and masticatory difficulties caused by interference between the displaced zygomatic arch and mandible are strong indicators for surgical intervention [9, 11]. Although several treatment algorithms exist, no uniform consensus has been established [1]. According to Raghoebar et al., treatment options include expectant management, closed reduction without fixation, and open reduction with or without fixation at one or more buttresses [1]. Surgical treatment aims to restore facial symmetry by reconstructing midfacial width, height, and projection [1–4]. Following accurate reduction, stability must be maintained through sufficient osteosynthesis to prevent functional and aesthetic sequelae [2, 6].

Various surgical approaches have been described [7]. The ideal approach should maximize accuracy of reduction while minimizing nerve and soft tissue injury to achieve both functional and aesthetic success [7]. Closed reduction is preferred when there is enough bone support between fragments and local muscle traction can maintain stability post-reduction. Despite concerns about limited visualization of the fracture site, the use of intraoperative 3D imaging addresses this limitation by improving visualization and accuracy of reduction [1]. Proponents of open reduction without fixation emphasize direct visualization and adequate reduction through periosteal elevation [1]. For unstable, multifragmentary fractures, most authors advocate open reduction and internal fixation as the most reliable approach, allowing better mobilization and vector-controlled reduction [2, 4, 6, 9, 12]. The use of 3D virtual planning, computer-aided design, and patient-specific templates further enhances reduction accuracy, reduces invasiveness, and shortens operative time [3, 13–17].

There is still no consensus on the minimal fixation required for stability, leaving the decision to surgeon discretion [1, 2, 4–7, 12, 18–25]. Many authors recommend determining fixation points based on fracture severity. Several clinical and biomechanical studies have supported 2- or 3-point fixation over 1-point fixation to improve postoperative malar projection and stability under masticatory loading [5, 18, 20, 22–25].

However, the growing demand for minimally invasive techniques with low wound complication and scarring risk has renewed interest in 1-point fixation for selected cases [6, 7, 19, 21, 26]. One rationale for this approach is the temporary reduction in masseter muscle force during the first 4–6 months postoperatively, which may lower the risk of delayed displacement [27]. Nevertheless, this notion is debated. Raghoebar et al. in his meta-analysis emphasized that masticatory muscle activity can exert significant stress on the zygomaticomaxillary complex, potentially causing postoperative asymmetry or dystopia, and noted that many studies insufficiently account for this factor [1]. The ideal fixation point also remains controversial, as scarring and exposure to different stress vectors influence stability. Most surgeons, however, prefer fixation at the zygomaticomaxillary buttress because it is less exposed to stress, provides rigid fixation through alignment with natural force vectors, and results in fewer palpable or visible scars than infraorbital or frontozygomatic buttress fixation [2, 4, 6, 7, 12, 18].

Most authors recommend 1-point fixation in non-comminuted ZMC fractures [2, 6, 21]. At our Level I trauma centre, we routinely perform 1-point fixation at the zygomaticomaxillary buttress for all patterns of ZMC fractures, regardless of the degree of displacement. The intraoperative use of 3D imaging provides us with immediate feedback on reduction accuracy, allowing for intraoperative decision-making regarding the need for corrective secondary reduction attempts and potential extension to more than one fixation points. Demonstrating the surgical accuracy of our concept could encourage other surgeons to consider 1-point fixation in comminuted ZMC fractures as well.

The primary aim of this study was to compare the intraoperative accuracy of repositioning following osteosynthesis with 1-point fixation versus multiple-point fixation in unilateral fractures of the ZMC complex. Our hypothesis was that the proposed approach would lead to adequate outcomes in terms of reconstructed malar dimensions and intraorbital volume. The secondary aim was to evaluate associations between trauma- and procedure-specific factors and the surgical outcomes of both fixation methods. A tailored, evidence-based approach — balancing clinical expertise with patient-centered factors — could help achieve optimal outcomes in the management of ZMC fractures.

## Methods

This retrospective single-centre observational study included all patients with unilateral fractures of the ZMC treated at the Department of Oral and Plastic Maxillofacial Surgery, Military Hospital Ulm. The study ranged from March 2019 and December 2024. Patient records were retrieved from electronic hospital databases.

Ethical approval was obtained from the Ethics Committee of the University of Ulm, Germany (approval reference: 203/24; approval date: 09. July 2024). The study was conducted in accordance with the Declaration of Helsinki (1964 and its later amendments).

Inclusion criteria were: (1) patients of all ages; (2) radiologically confirmed unilateral fractures of the ZMC with preoperative CT imaging; (3) intraoperative 3D scan; (4) fracture reduction and osteosynthesis using 1-point fixation through Keen’s intraoral approach or multiple-point fixation through combined intraoral and extraoral approach.

Exclusion criteria included (1) patients with bilateral ZMC fractures; (2) patients presenting with isolated zygomatic arch fractures and/or additional midfacial fracture patterns; (3) lack of preoperative CT or intraoperative 3D imaging; (4) incomplete CT scan; (5) non-dislocated ZMC fractures treated conservatively; (6) dislocated ZMC fractures managed conservatively due to patient-related factors (e.g., advanced age, dementia, or refusal of surgery); and (7) incomplete medical records.

### Patient screening

All patients underwent a clinical assessment, radiological imaging and laboratory tests upon admission to the emergency department. The clinical examination included general and trauma-related medical history, a general examination and a specific maxillofacial examination for clinical signs ZMC fractures, as well as interdisciplinary specialized ophthalmological assessment. All patients were evaluated by board-certified colleagues in oral and maxillofacial surgery and ophthalmology. Our standard protocol for initial diagnosis included panoramic radiographs and a multiplanar 3D CT scan of the facial skeleton. The treatment method (surgical or conservative) was individually determined by a board-certified oral and maxillofacial surgeon.

### Surgical treatment

Patients were treated according to standardized departmental protocols through a Keen’s intraoral approach. Preoperatively, all patients without contraindications received a single intravenous dose of 3 g ampicillin-sulbactam (Unacid^®^, Pfizer Pharma GmbH) and 250 mg of prednisolone (Solu-Decortin^®^, Merck Serono GmbH). In cases of penicillin allergy, patients received a single intravenous dose of 600 mg Clindamycin.

Preoperatively, the patient was positioned on an operating table with a carbon fibre head section, providing optimal radiolucency for intraoperative 3D imaging. Following intraoral germ reduction using an antiseptic mouthwash, articaine with epinephrine (Ultracain^®^ D-S forte, Septodont) was infiltrated into the surgical field. After extra- and intraoral disinfection, a gingivobuccal incision was performed 1 cm above the attached gingiva. The incision was then deepened through the buccinator muscle straight to the anterior maxillary wall until the periosteum was identified. Following subperiosteal dissection, the mucoperiosteal flap was elevated, revealing fracture site. A zygoma hook elevator was carefully inserted behind the infratemporal surface of the zygomatic bone to reduce the fractured segments into correct anatomical position. Superior, lateral and anterior forces were applied for reduction. Simultaneously, the contralateral hand was used to stabilize the patient’s head and facilitate accurate reduction of the fracture. An audible click, restoration of cheek fullness, and palpation of the normal contour of the zygomatic body and infraorbital rim served as intraoperative indicators confirming adequate fracture reduction. A single-point fixation at the zygomaticomaxillary buttress was then performed with one or more miniplates (0.7–1 mm thickness) and monocortical screws (4 mm in length, 1.5 mm diameter; MatrixMIDFACE™ Plating System, DePuy Synthes/Johnson & Johnson MedTech, Massachusetts, USA).

The intraoperative 3D imaging (Cios Spin, Siemens Healthineers, Erlangen, Germany) CBCT scan (Cone Beam Computed Tomography) and data acquisition were performed to verify fracture reduction and plate positioning.

**Fig. 1:**
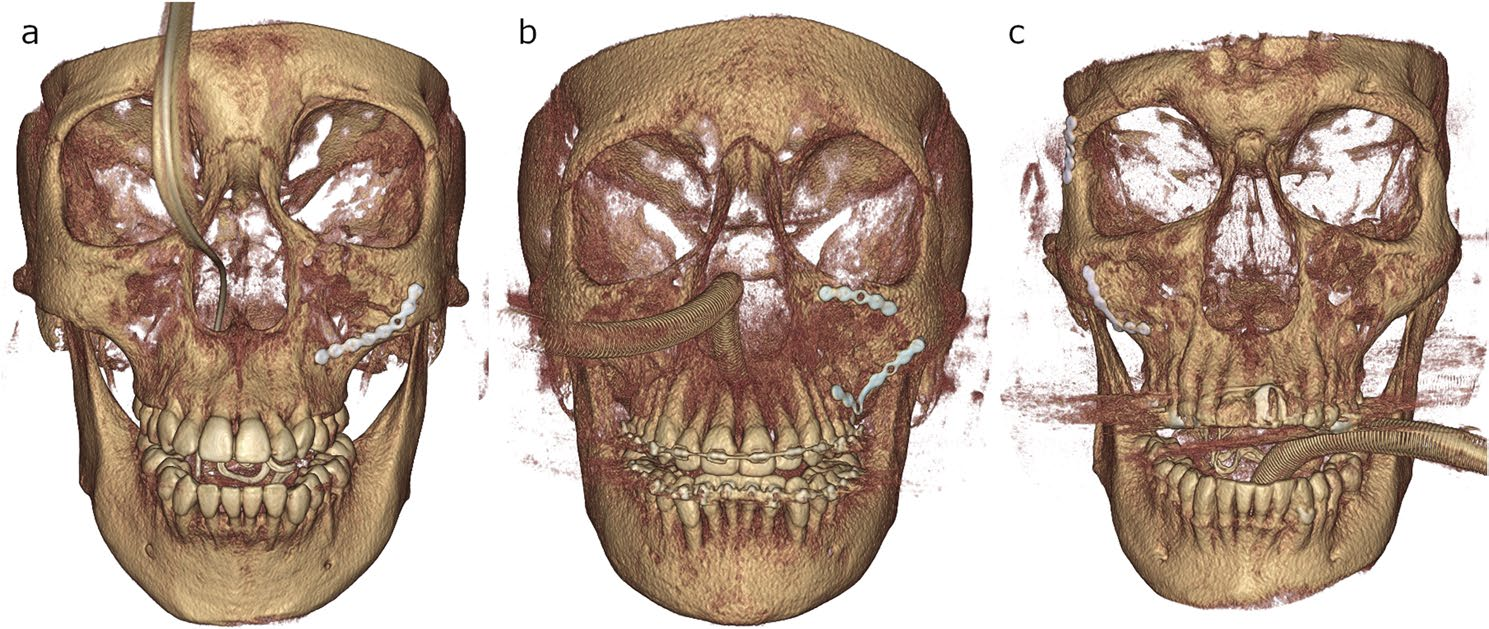
Postoperative view after repositioning and osteosynthesis of unilateral zygomaticomaxillary fractures: (**a**) 1-point fixation, (**b**) 2-point fixation (zygomaticomaxillary buttress and lateroorbital rim), (**c**) 2-point fixation (zygomaticomaxillary buttress and infraorbital rim)

If malreduction was identified, further reduction manoeuvres were performed. When intraoperative control continued to demonstrate insufficient anatomical alignment, the decision to add further fixation points (e.g., infraorbital rim via a transconjunctival approach and/or at the lateral orbital rim through a blepharoplasty incision) was made (Fig. 1a-c). This decision was based on intraoperative findings and the operating surgeon’s clinical judgment, taking into account fracture comminution, stability after reduction, and the expected functional and aesthetic outcome. Upon confirmation of satisfactory fixation and occlusion, the surgical wound was closed using continuous 4 − 0 Vicryl sutures (Ethicon, Sint-Stevens-Woluwe, Belgium).

### Postoperative cara and follow-up

All patients were discharged at the first postoperative day and were instructed to maintain a soft diet for four weeks. Postoperative instructions included the use of prescribed analgesics and nasal decongestants, along with the recommendation to avoid nasal insufflation (e.g., sniffing) for a period of two weeks to support optimal wound healing. Postoperative care did not include the administration of prophylactic antibiotics. Throughout the follow-up period, all patients underwent clinical assessment by independent observers.

### Data collection

Patient data were anonymized prior to analysis and included: age at injury; gender; trauma aetiology; fracture pattern; clinical symptoms (e.g., occlusal disorder, infraorbital nerve function); trauma-to-surgery interval, intraoperative details (e.g., type of osteosynthesis, need for intraoperative revision); treatment outcomes (e.g., accuracy of fracture repositioning); follow-up period.

Radiological data regarding fracture characteristics and dental status were collected from CT and CBCT scans which were taken for pre- and intraoperative diagnostics. All preoperative CT scans followed the standard institute protocol and were interpreted by two board-certified radiologists. The intraoperative CBCT scans were interpreted by the attending surgeon. The patients received no further radiation exposure for this study.

### Measurements zygomatic complex

The dimensions of the zygomatic complex were measured based on CT scans of the patients stored on the hospital’s server. CT scans from other hospitals were obtained and imported into the database of our radiology department. All intraoperative CBCT scans were obtained by the surgical teams of our department, following standardized imaging protocols.

**Fig. 2:**
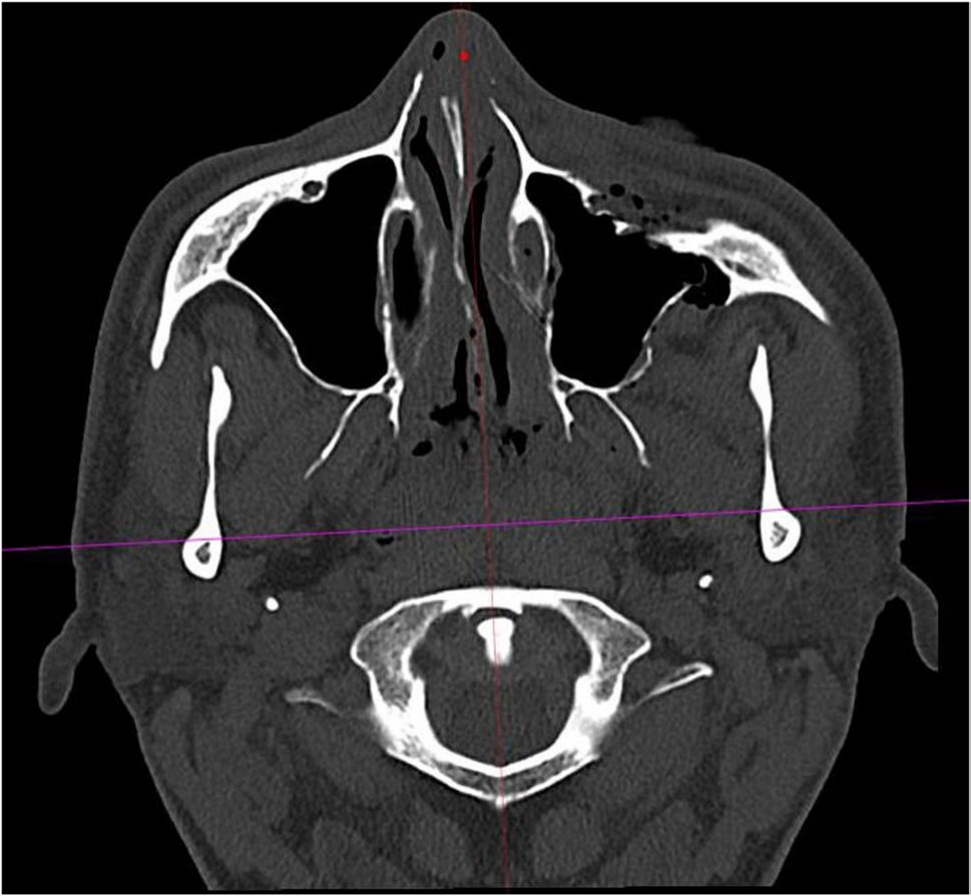
Initial symmetrical display of the two ascending mandibular rami

**Fig. 3:**
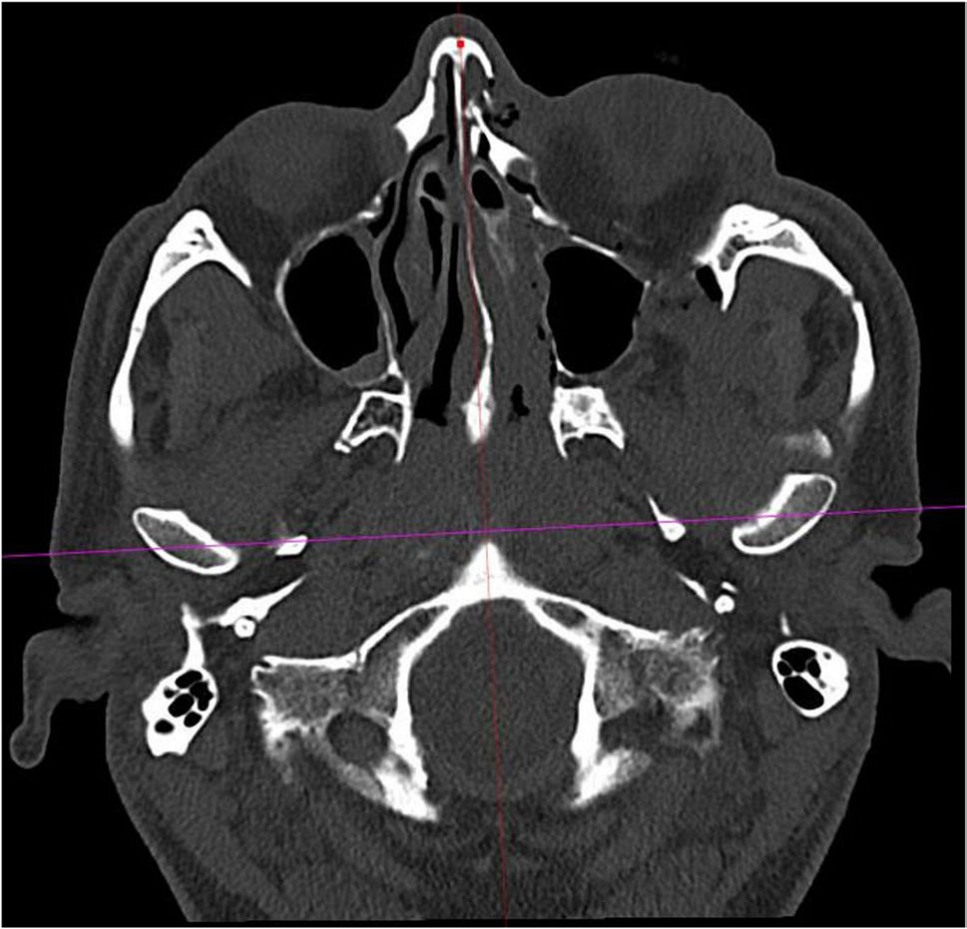
A line connecting the centers of the two mandibular condyles served as the first of the two reference lines for measurements

**Fig. 4:**
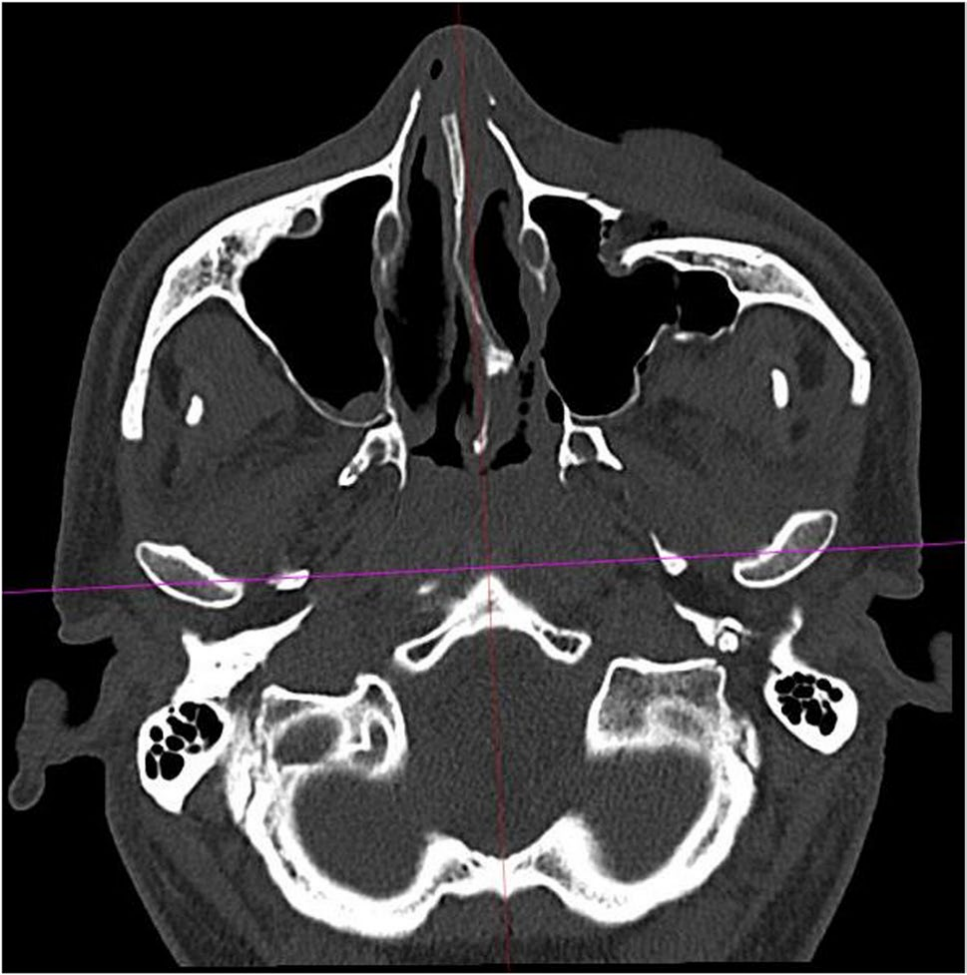
A second sagittal reference line was placed orthogonally to the first, passing through the anterior margin of the foramen magnum and the sphenoidal rostrum or vomer as anatomical reference structures

**Fig. 5:**
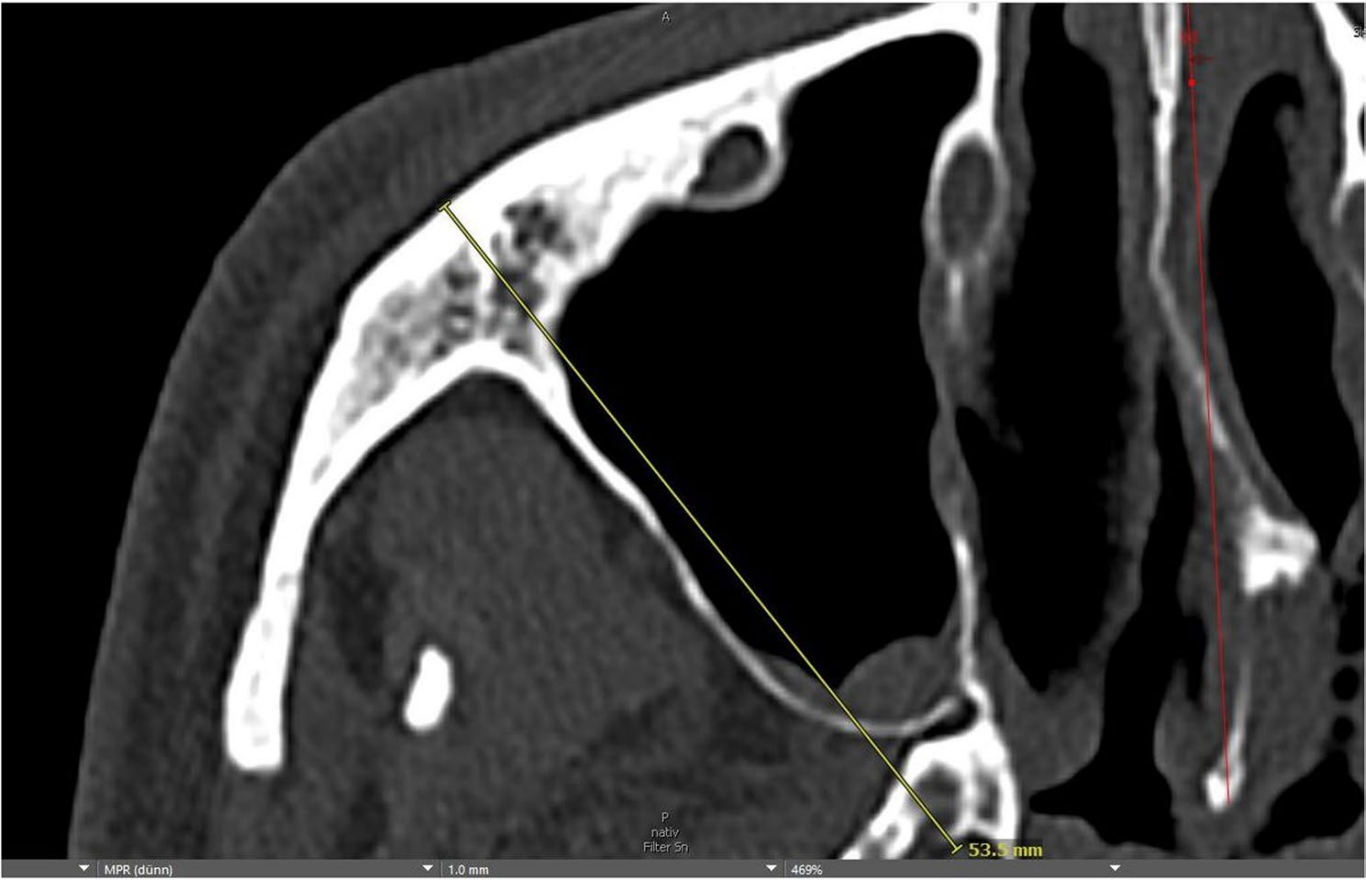
The zygomatic prominence was designated as the aesthetic reference point

**Fig. 6:**
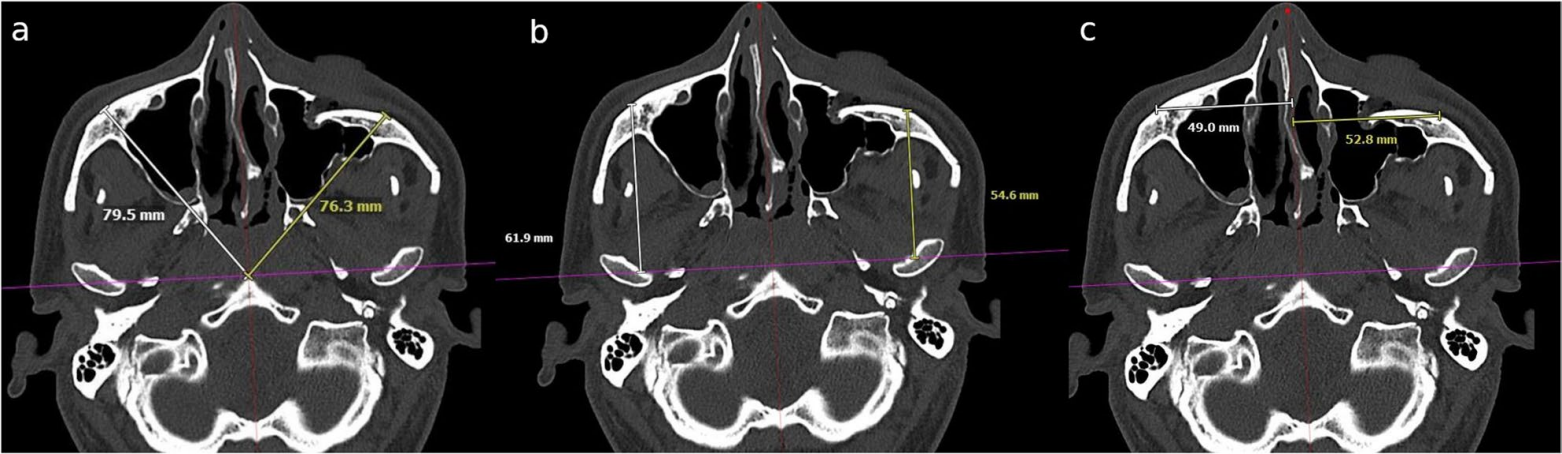
From zygomatic prominence, three distances were measured: (**a**) a diagonal to the intersection of the two reference lines, (**b**) the malar height up to the axial reference line, and (**c**) the intermalar width up to the sagittal reference line

Initially, preoperative CT scans were measured in the midface bone window in the axial plane. Due to variations in patient positioning during the scan, the two ascending mandibular rami were displayed symmetrically using software tools (Visage^®^ 7 Enterprise Imaging Platform, Berlin, Germany) (Fig. 2). The mandibular condyles were then displayed symmetrically in their most retral position. A line connecting the centres of the two mandibular condyles served as the first of two reference lines for measurements (Fig. 3). The image was then rotated around this line until the zygomatic prominences were visible on both sides ensuring a symmetrical representation. A second sagittal reference line was placed orthogonally to the first, passing through the anterior margin of the foramen magnum and the sphenoidal rostrum or vomer as anatomical reference structures (Fig. 4). The skull was divided into two mirror-symmetrical halves. To minimize the effect of individual anatomical asymmetry (e.g., septal deviation, vomer angulation), reference lines were placed using consistent bony landmarks. Minor deviations were corrected by applying an averaging method between the left and right landmarks, thereby standardizing orientation across patients rather than aiming for absolute mirror symmetry. This ensured reproducibility of the measurement protocol while acknowledging natural inter-individual differences. For the optimal spatial presentation of the zygomatic prominence, measurements were taken from the unaffected zygoma side, as well as the fractured and repositioned zygoma side, in both the preoperative CT scan and the intraoperative C-arm CBCT scan. Three measurements ​​were taken for each side of the skull. The zygomatic prominence mentioned earlier was designated as the aesthetic reference point (Fig. 5). From this point, three distances were measured: a diagonal to the intersection of the two reference lines, the malar height up to the axial reference line, and the intermalar width up to the sagittal reference line (Fig. 6a-c). For the latter two distances, lines were drawn orthogonally to the respective reference lines. All measurements were recorded in millimeters (mm) with one decimal place and documented photographically.

### Asymmetry index

**Fig. 7:**
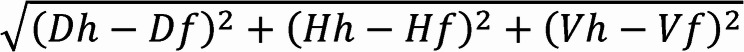
Asymmetry Index. Abbreviations: Dh = Diagonal healthy side; Df = Diagonal fractured side; Hh = Malar height, healthy side; Hf = Malar height fractured side; Vh = Vertical length healthy side; Vf = Vertical length fractured side

To quantify the position of the dislocated zygomatic body before and after fracture reduction, an asymmetry index was utilized following the formula [28–30]:

Dh and Df represent the diagonals of the healthy and fractured side, Hh and Hf indicate the malar heights, and Vh and Vf denote the intermalar widths. The asymmetry index was calculated for both the preoperative and intraoperative scans, with results expressed to three decimal places. Additionally, the difference in the asymmetry index between the two images was calculated Fig 7.

### Intraorbital volume

The orbital volume was calculated using Brainlab Planning Software (Brainlab AG, Munich, Germany). A uniform measurement protocol was applied throughout to maintain consistency across all evaluations. Preoperative multislice CT scans and intraoperative CBCT scans were imported into the iPlan-CMF Planning Software (Brainlab) through the hospital’s internal database (Visage^®^ 7 Enterprise Imaging Platform, Berlin, Germany). CT scans used for measuring the zygomatic complex were also applied for orbital measurement. The affected side was measured on both the preoperative CT scan and the intraoperative CBCT scan, while the unaffected contralateral side was measured on the preoperative CT scan as a reference. Patient cases where the orbits were not fully visible in either scan were excluded from the study, impacting 27 patient cases.

Through semi-automatic volume measurement, the orbit was automatically detected in most cases using preconfigured algorithms based on planimetric volumetry. Manual post-processing adjustments were performed using appropriate editing tools within the 3D multiplanar reconstruction view. In cases where the software produced inaccurate orbital volume calculations, manual redefinition of the orbital boundaries was carried out.

**Fig. 8:**
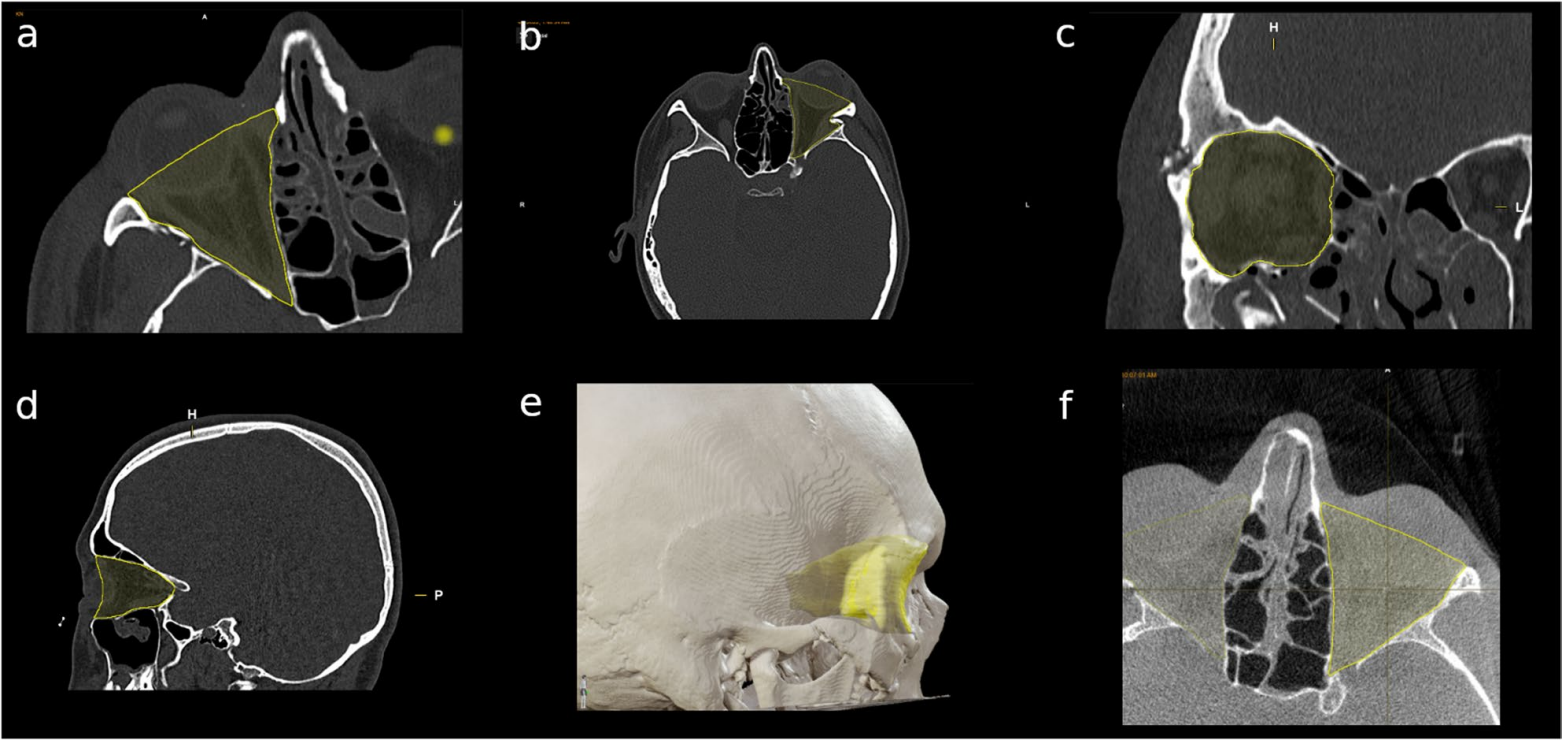
Calculation of the intraorbital volume preoperatively and postoperatively. **a**-**b** Axial view: The anterior orbital border was defined by drawing a straight line between the medial and lateral bony borders of the orbit. In areas with fractures on the infraorbital rim or the anterolateral orbital floor, the edges of the fractured bone portions were connected with a straight line to define the boundary in that area, (**c**) Coronal view: Drawing the intraoorbital volume aligned with the bony frame, (**d**) Sagittal view: The anterior border was defined by a parabolic curve between the cranial and caudal bony borders of the orbit, following the parabolic shape of the medial and lateral bony borders, (**e**) 3D rendering of the intraorbital volume, (**f**) Axial view of the intraorbital volume after fracture reduction

The anterior orbital border was defined by drawing a straight line between the medial and lateral bony borders of the orbit in the axial view. In the sagittal view, the anterior border was defined by a parabolic curve between the cranial and caudal bony borders of the orbit, following the parabolic shape of the medial and lateral bony borders. In coronal reconstruction, care was taken to verify that the delineated orbital volume corresponded precisely to the anatomical contours of the bony orbit. Posteriorly, the orbit expanded in both axial and sagittal planes in a funnel-shaped manner along the bony contours, gradually narrowing and straightening in the region of the optic canal and the superior orbital fissure. Other physiological orbital foramina (e.g., inferior orbital fissure, foramen rotundum) were approximated as flat planes to standardize the orbital volume assessment. Where fractures affecting the infraorbital rim or anterolateral orbital floor, their bony edges were connected using a linear interpolation to define the orbital boundary for volume calculation. Soft tissue prolapses resulting from orbital wall fractures were accounted for and included in the overall volume measurements. Following this systematic procedure, all CT slices were adjusted in multiple planes and thoroughly checked. The orbital volume was then calculated in cubic centimetres (cm^3^) using iPlan CMF™ software (Fig. 8).

### Reliability

Data reliability was assessed for the measurements of the zygomatic complex, as well as for the registration and segmentation steps involved in intraorbital volume analysis. To evaluate intraobserver reliability, the same observer repeated the measurement procedures twice using identical CT views and the same segmented 3D models across all patients. However, subsequent statistical analysis did not demonstrate sufficient inter- and intraobserver reliability to support the robustness of the measurement methods employed in this study.

### Study variables

Independent variables included trauma- and procedure-specific factors.

1. Radiological variables of the ZMC fracture [31]:
  (A) Zingg classification of zygomatic fractures (A2,3/B/C).
2. Surgical variables
  (A) Trauma-to-surgery interval (in days).
  (B) Surgeon level (resident/consultant).

### Outcome measures

Primary outcome: The accuracy of fracture repositioning was evaluated by comparing the final intraoperative position after osteosynthesis, as visualized on CBCT, with the preoperative position on the initial CT scan in both fixation groups (1-point fixation vs. multiple-point fixation). This calculation was based on three parameters that determined the dependent variables:

- *Asymmetry Index* = Quantification of the position of the dislocated zygomatic body before and after fracture reduction [28–30].
- *Malar width difference* = Difference of the malar width between the healthy and the fractured side preoperatively and intraoperatively after reduction.
- *Malar height difference* = Difference of the malar height between the healthy and the fractured side preoperatively and intraoperatively after reduction.
- *Malar diagonal difference* = Difference of the malar diagonal dimension between the healthy and the fractured side preoperatively and intraoperatively after reduction.
- *Difference in intraorbital volume =* Difference of the infraorbital volume between the healthy and the fractured side preoperatively and intraoperatively after reduction.

### Statistical analysis

Data was centralized in electronic format using Microsoft Excel (Microsoft Corporation, Redmond, USA) and analyzed descriptively. Statistical analysis was performed using R Version 4.4.3 (R Core Team). Descriptive statistics summarized baseline characteristics. Categorical variables were presented as counts and percentages. Age, trauma-to-surgery interval and follow-up interval were reported using mean ± SD or range.

The Shapiro-Wilk test was applied to assess the normality of data distribution. Spearman correlation analysis was used to evaluate associations between continuous predictor variables and postoperative outcomes. The Wilcoxon rank-sum test was employed to analyze associations between dichotomous predictor variables and postoperative outcomes, while ANOVA was used for non-dichotomous nominal variables. Univariate subgroup comparisons were conducted using either an independent paired t-test or the Wilcoxon test, depending on data distribution. A p-value ≤ 0.05 was considered statistically significant.

## Results

### Demographic distribution

A total of 62 patients with 62 unilateral ZMC fractures were included in the analysis. The majority were male (45/62; 72.6%), resulting in a male-to-female ratio of 2.64:1. The mean age at the time of injury was 43.74 ± 18.65. years (range: 12–88 years).

### Etiology

Overall, interpersonal violence was the leading cause of injury (*n* = 16; 25.8%), followed by bicycle accident (*n* = 15; 24.2%), tripping falls (*n* = 7; 11.3%), falls due to internal cause (*n* = 7; 11.3%) and sport (*n* = 7; 11.3%). Less frequent were traffic accidents (*n* = 2; 3.2%) and accidents of other aetiologies (*n* = 8; 12.9%).

### ZMC fracture patterns

Out of the 62 ZMC fractures, 28 (45.2%) involved the right and 34 (54.8%) involved the left side. The paranasal buttress was affected in 11 cases (17.7%). According to the Zingg classification, 20 fractures (32.3%) were classified as Type A, 25 (40.3%) as Type B, and 17 (27.4%) as Type C. Within the Type A group, 5 fractures were classified as A2 (isolated lateroorbital rim fractures) and 12 as A3 (isolated infraorbital rim fractures). Isolated zygomatic arch fractures (A1) were excluded by study design. No separate statistical analysis was performed for the Type A subgroups.

### Surgical treatment

Forty-two patients underwent treatment with 1-point fixation, while 20 patients received multiple-point fixation. The average trauma-to-surgery interval was 5.02 ± 3.27 days (range: 0–13 days).

Within the 1-point fixation group (group 1), 30 cases (71.4%) received a single osteosynthesis plate at the zygomaticomaxillary buttress. In 10 cases (23.8%), two plates were placed, and in 2 cases (4.8%), three plates were placed.

In the multiple-point fixation group (group 2), 11 cases (55%) were treated with two osteosynthesis plates (6 cases involving the zygomaticomaxillary buttress and infraorbital rim and 5 cases involving the zygomaticomaxillary buttress and lateroorbital rim). Three-point fixation (zygomaticomaxillary buttress, infraorbital rim and lateroorbital rim) was employed in 7 cases (35%), while 2 cases (10%) required four-point fixation.

In both fixation groups, 3 cases (1-point fixation = 7.1%; multiple-point fixation = 15%) underwent intraoperative revision after intraoperative 3D imaging due to inadequate fracture reduction.

The mean follow-up period was 5.47 ± 4.60 months.

### Analysis

**Fig. 9:**
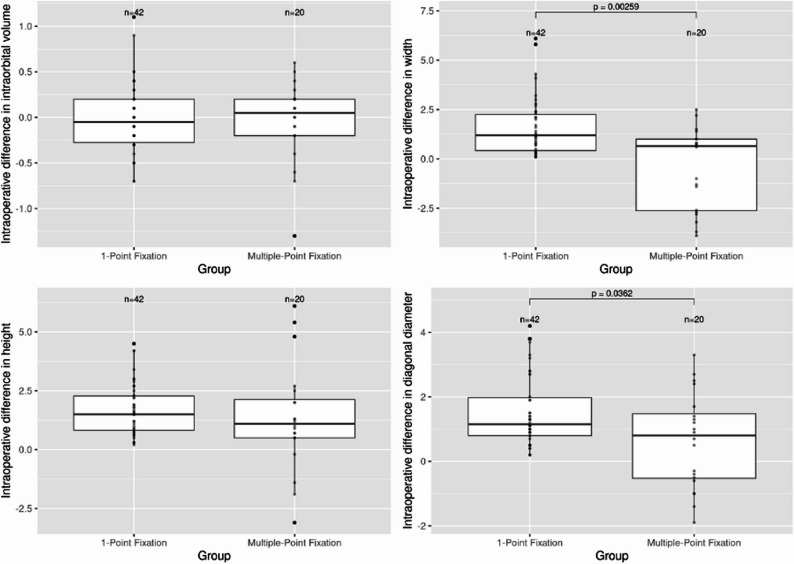
Intraoperative surgical outcomes in terms of malar geometry: comparison of group 1 and group 2

**Fig. 10:**
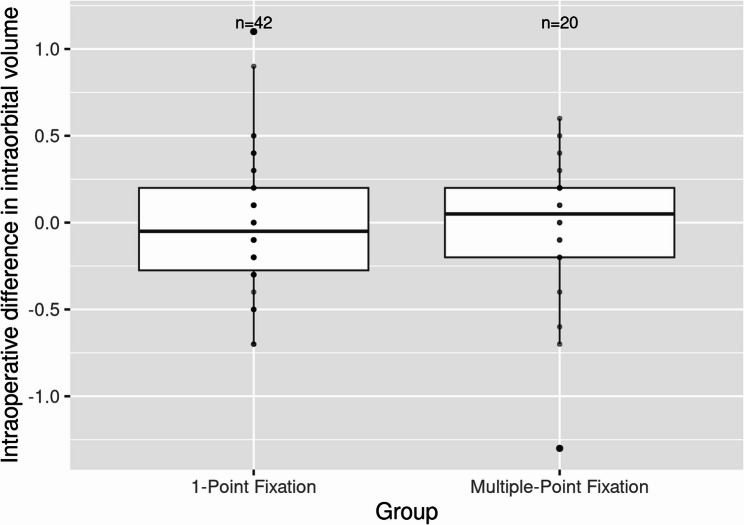
Intraoperative surgical outcomes in terms of intraorbital volume: comparison of group 1 and group 2

Preoperatively, the means of asymmetry index, malar width difference, malar height difference, malar diagonal difference and intraorbital volume difference of group 1 were 4.96 ± 2.96, 2.51 ± 1.95 mm, 2.54 ± 2.05 mm, 2.75 ± 2.25 mm, and 0.86 ± 0.96 cm^3^, respectively, and those of group 2 were 6.58 ± 3.13, 1.55 ± 2.68 mm, 3.96 ± 2.43 mm, 3.93 ± 2.63 mm, and 1.17 ± 1.12 cm3. The preoperative malar height difference was significantly higher in group 2 (*p* = 0.02) (Table 1; Figs. 9 and 10). The intraoperative malar width difference and malar diagonal difference were significantly greater in group 1 compared to the other groups (*p* = 0.003 and *p* = 0.036, respectively). No statistically significant difference in intraorbital volume was observed between the two fixation groups. All variables demonstrated highly statistically significant differences between preoperative and postoperative measurements within each group (Table 2).

**Table 1:** Surgical outcomes: comparison of group 1 and group 2

Preoperative variables		1-point fixation		multiple-point fixation	
	n	Mean ± SD	n	Mean ± SD	*p* value**
Asymmetry Index*	42	4.957 ± 2.961	20	6.577 ± 3.133	0.056
Malar width difference	42	2.51 ± 1.92 mm	20	1.55 ± 2.68 mm	0.35
Malar height difference	42	2.54 ± 2.05 mm	20	3.96 ± 2.43 mm	**0.02**
Malar diagonal difference	42	2.76 ± 2.26 mm	20	3.94 ± 2.63 mm	0.054
Intraorbital volume difference	42	0.86 ± 0.96 cm^3^	20	1.17 ± 1.12 cm^3^	0.23
Intraoperative variables					
Asymmetry Index*	42	3.069 ± 1.707	20	3.224 ± 1.793	0.763
Malar width difference	42	1.58 ± 1.47 mm	20	−0.47 ± 2.07 mm	**0.003**
Malar height difference	42	1.71 ± 1.11 mm	20	1.35 ± 2.27 mm	0.299
Malar diagonal difference	42	1.53 ± 1.12 mm	20	0.63 ± 1.48 mm	**0.036**
Intraorbital volume difference	42	−0.01 ± 0.381 cm^3^	20	−0.04 ± 0.45 cm^3^	0.795

*Abbreviations*: *SD* Standard Deviation

*No unit of measurement

**t-test;* p* value ≤ 0.05 = statistically significant (boldface)

**Table 2:** Surgical outcomes: comparison of preoperative and intraoperative variables within each group

1-point fixation		preoperative		intraoperative	
	n	Mean ± SD	n	Mean ± SD	*p* value**
Asymmetry Index*	42	4.957 ± 2.961	42	3.069 ± 1.707	**< 0.001**
Malar width difference	42	2.51 ± 1.92 mm	42	1.58 ± 1.47 mm	**0.007**
Malar height difference	42	2.54 ± 2.05 mm	42	1.71 ± 1.11 mm	**0.008**
Malar diagonal difference	42	2.76 ± 2.26 mm	42	1.53 ± 1.12 mm	**< 0.001**
Intraorbital volume difference	42	0.86 ± 0.96 cm^3^	42	−0.01 ± 0.38 cm^3^	**< 0.001**
**Multiple-point fixation**					
Asymmetry Index*	20	6.57 ± 3.13	20	3.22 ± 1.79	**< 0.001**
Malar width difference	20	1.55 ± 2.68 mm	20	−0.47 ± 2.07 mm	**0.004**
Malar height difference	20	3.96 ± 2.43 mm	20	1.35 ± 2.27 mm	**< 0.001**
Malar diagonal difference	20	3.94 ± 2.63 mm	20	0.63 ± 1.48 mm	**< 0.001**
Intraorbital volume difference	20	1.17 ± 1.12 cm^3^	20	−0.04 ± 0.45 cm^3^	**< 0.001**

*Abbreviations*: *SD* Standard Deviation

*No unit of measurement

**t-test; *p* value ≤ 0.05 = statistically significant (boldface)

Table 3 presents the surgical outcomes (intraoperative asymmetry index, intraorbital volume difference between healthy and fractured side of zygoma, intraorbital volume difference between healthy and reduced side of zygoma) for both fixation groups, in association with the predictor variables. Univariate analysis revealed that a trauma-to-surgery interval of 3–7 days was significantly associated a better repositioning result in terms of intraorbital volume difference after multiple-point fixation (*p* = 0.03).

**Table 3:** Distribution of predictor variables in correlation with surgical outcomes in group 1 and group 2

	*1-point fixation*									*Multiple-point fixation*								
		Asymmetry Index Intraoperatively			Intraorbital Volume Difference (healthy-fractured)			Intraorbital Volume Difference (healthy-reduced)			Asymmetry Index Intraoperatively			Intraorbital Volume Difference (healthy-fractured)			Intraorbital Volume Difference (healthy-reduced)	
Fracture classification (1)	n	Mean ± SD	p value*	n	Mean ± SD (cm^3^)	p value*	n	Mean ± SD (cm3)	p value*	n	Mean ± SD	p value*	n	Mean ± SD (cm^3^)	p value*	n	Mean ± SD (cm^3^)	p value*
Type A	17	2.84 ± 1.28	0.3	17	0.59 ± 0.53	0.23	17	−0.04 ± 0.28	0.91	3	3.76 ± 2.13	0.27	3	2.10 ± 0.44	0.14	3	−0.10 ± 0.10	0.76
Type B	15	2.97 ± 1.83		15	1.10 ± 1.10		15	0.05 ± 0.37		10	3.51 ± 2.21		10	1.12 ± 0.90		10	0.04 ± 0.32	
Type C	10	3.59 ± 2.18		10	0.98 ± 1.23		10	−0.04 ± 0.54		7	2.58 ± 0.74		7	0.84 ± 1.46		7	−0.13 ± 0.68	
Trauma-to-surgery interval	n			n			n			n			n			n		
0–2 days	12	3.27 ± 1.76	0.76	12	0.91 ± 1.04	0.93	12	−0.05 ± 0.44	0.86	4	4.56 ± 2.93	0.14	4	1.00 ± 0.61	0.13	4	0.18 ± 0.05	**0.03**
3–7 days	21	3.11 ± 1.89		21	0.89 ± 1.05		21	−0.01 ± 0.38		10	2.52 ± 1.04		10	0.78 ± 1.33		10	−0.29 ± 0.49	
> 7 days	9	2.71 ± 1.22		9	0.76 ± 0.64		9	0.04 ± 0.33		6	3.51 ± 1.61		6	1.93 ± 0.58		6	0.23 ± 0.28	
Surgeon level	n			n			n			n			n			n		
Resident	27	2.73 ± 1.50	0.082	27	0.91 ± 0.88	0.65	27	−0.03 ± 0.38	0.65	7	3.31 ± 1.73	0.89	7	1.30 ± 1.02	0.71	7	−0.03 ± 0.35	0.94
Consultant	15	3.68 ± 1.92		15	0.77 ± 1.10		15	0.03 ± 0.39		13	3.18 ± 1.89		13	1.10 ± 1.21		13	−0.05 ± 0.51	

*Abbreviations*: *SD* Standard Deviation

*ANOVA univariate analysis;* p* value ≤ 0.05 = statistically significant (boldface)

## Discussion

This retrospective analysis of 62 patients with unilateral zygomaticomaxillary complex (ZMC) fractures highlights the relevance of fixation strategy and surgical timing on intraoperative precision of fracture reduction using either 1-point or multiple-point fixation. To our knowledge, this is among the few studies systematically comparing 1-point and multiple-point fixation regarding detailed volumetric and topographic facial symmetry parameters derived from intraoperative 3D imaging.

Briefly summarized, our findings demonstrate that multiple-point fixation is associated with a significantly intraoperatively improved reduction in malar width difference and malar diagonal difference. This aligns with the conclusions of Eski et al., who found that two- or three-point fixation strategies offer enhanced biomechanical stability and midfacial contour restoration, particularly in comminuted or displaced fractures [32]. In contrast, intraorbital volume measurements showed no significant differences between the two fixation groups. An early intervention (within 3–7 days post-trauma) was identified as a favorable factor for achieving improved intraorbital volume symmetry, particularly in the multiple-point fixation group.

In line with previous epidemiological data, ZMC fractures were more prevalent in males and showed a wide age distribution, with interpersonal violence and bicycle accidents as leading causes of injury [33, 34]. This reflects previously reported demographic trends in maxillofacial trauma, where young adult males are disproportionately affected by midfacial fractures, often due to assault or traffic-related trauma [31].

### Interpretation and clinical relevance

Our findings support the growing notion that fixation strategy in ZMC fractures should be carefully tailored to the fracture complexity and displacement pattern. The increased malar asymmetry observed in the single-point fixation group reflects the limitations of relying on a single vector of control when repositioning a structure as complex and three-dimensionally oriented as the zygoma. Particularly in displaced fractures, multiple-point fixation offers the benefit of distributing stabilization forces across several anatomical buttresses, enabling better control of torque, rotation, and medialization of the zygomatic bone.

Interestingly, the intraorbital volume—an important marker for orbital symmetry and enophthalmos risk—was not significantly different between the two groups. This implies that the accuracy of orbital volume restoration may not be solely dependent on the number of fixation points but may instead reflect the quality of the initial reduction, the anatomical integrity of the orbital walls, and the use of intraoperative 3D imaging guidance.

The timing of surgical intervention appears to play a significant role in optimizing intraoperative results. The positive association between surgical timing (within 3–7 days) and better volume correction suggests a biological and practical window for optimal fracture manipulation. Within this timeframe, periorbital edema typically recedes, improving surgical visibility and tactile feedback, while fibrous healing has not yet compromised mobility of fracture segments.

### Single-point fixation as a viable option

A growing body of literature supports the clinical viability of single-point fixation for selected ZMC fractures—particularly those that are non-comminuted and minimally displaced [4–6, 12, 21, 30]. The rationale for this approach lies in its reduced surgical morbidity, shorter operative time, and avoidance of additional incisions that may lead to visible scarring or nerve injury.

Shokri et al. presented one of the most comprehensive long-term studies on this topic, analyzing 162 patients over 20 years who underwent single-point fixation at the zygomaticomaxillary buttress [21]. Despite using only one fixation point, complication rates were low (one tooth loss, eight infections), and no cases of malar asymmetry, enophthalmos, or hypoglobus required revision surgery—suggesting that in selected cases, single-point fixation can provide adequate stability without compromising function or aesthetics. Similarly, Kim et al. reported significant postoperative improvement in zygomatic alignment using 3D CT in 29 patients treated with single-point zygomaticomaxillary buttress fixation. Vertical and horizontal displacement were effectively corrected, with no revisions required. However, they noted that horizontal stability was reduced in comminuted fractures involving the buttress itself, underscoring the importance of patient selection [6]. iomechanical findings from Refahee et al. further support these clinical results: their finite element analysis showed that zygomaticomaxillary buttress fixation produced the lowest stress concentrations and micromotion among single-point options, indicating superior mechanical stability [4]. Abu Dakir et al. reached similar conclusions in 30 patients treated via a transoral (Keen’s) approach, reporting excellent facial symmetry and occlusion without additional fixation points, and advocating this technique for aesthetic midface restoration [35].

While our study demonstrated that multiple-point fixation achieved greater intraoperative accuracy in malar width and projection, these anatomical advantages did not result in higher functional or aesthetic benefit. Intraorbital volume restoration—a marker of orbital symmetry and enophthalmos risk—did not differ significantly between groups, and minor residual asymmetries were clinically negligible. These findings align with Rahbin et al., who reported no difference in long-term satisfaction among one-, two-, and three-point fixation techniques in a CT-based follow-up of 136 ZMC fractures [36]. Even when radiological asymmetries were detected, they did not affect patient-perceived outcomes, reinforcing that absolute symmetry is not essential for functional or aesthetic success.

Therefore, despite the theoretical advantages of multiple-point fixation in achieving precise skeletal alignment, the current body of evidence—including our own findings—suggests that these anatomical improvements may not necessarily yield superior clinical outcomes in terms of function or facial aesthetics. As such, single-point fixation remains a highly valuable technique, particularly in straightforward fracture patterns, provided that careful patient selection, careful intraoperative assessment and proper reduction are ensured [7, 21, 37].

In clinical decision-making, the advantages of reduced surgical trauma, shorter operative duration, and fewer access-related complications should be weighed against the need for exact three-dimensional repositioning. When intraoperative imaging or navigation is available, these tools may help bridge the gap by verifying reduction accuracy in real time—enhancing the reliability of single-point fixation strategies even in borderline cases.

### Superiority of multiple-point fixation in complex fractures

Although 1-point fixation has been advocated for its effectiveness considering stability with minimal invasiveness, reduced complication rates and operative time, several studies have questioned its adequacy in restoring three-dimensional symmetry and stability. On the other hand, multiple-point fixation continues to be advocated for complex, comminuted, or significantly displaced fractures. Our findings suggest that while single-point fixation may suffice in restoring orbital volume, it may fall short in re-establishing the three-dimensional geometry of the zygomatic prominence.

Our findings demonstrate that intraoperative differences in malar width and diagonal measures were significantly greater in the 1-point fixation group and consequently, multiple-point fixation is associated with a significantly improved reduction in malar height difference, particularly in patients with more complex fracture configurations (Zingg Type B and C). These findings agree with existing literature emphasizing the biomechanical superiority of multiple-point fixation for restoring midfacial projection and orbital integrity [7, 32]. While the higher preoperative malar height discrepancy in the multiple-fixation group may reflect greater initial trauma severity, the favourable postoperative outcomes suggest that multiple-point fixation can effectively compensate for more extensive displacement [22]. The 1-point fixation provides limited control over three-dimensional spatial alignment, potentially resulting in suboptimal zygomatic positioning. Although no significant postoperative difference in orbital volume asymmetry was observed between groups, both strategies yielded significant improvements in symmetry and volume parameters, underscoring the overall efficacy of surgical treatment [1].

Lee et al. compared 1-point and 2-point fixation and found that dual fixation—particularly involving the zygomaticofrontal suture—offered superior restoration of malar height and better facial symmetry [5]. The authors also highlighted that the addition of an infraorbital rim plate may aid in addressing vertical dystopia [5]. Likewise, the meta-analysis by Neto et al. noted that although single-point fixation reduced operative morbidity, multiple-point approaches resulted in better long-term anatomical and functional outcomes in more severe ZMC fractures [2]. Zingg et al., in a seminal study of over 1000 cases, emphasized that incomplete or inadequate reduction of the lateral orbital complex often led to postoperative orbital volume increase, enophthalmos, and aesthetic dissatisfaction. Their classification of ZMC fractures underscores the importance of tailoring fixation techniques to the severity of displacement and fracture morphology [31].

### Surgical timing

A key contribution of this study is the analysis of surgical timing. Patients operated on within 3 to 7 days after trauma showed significantly better restoration of orbital volume in the multiple-point fixation group. This aligns with evidence suggesting that early intervention before significant fibrous consolidation facilitates better more accurate reductions and anatomical realignment and reduces the risk of late enophthalmos and asymmetry [38–40]. Delayed surgery has been associated with more challenging reductions and increased complication rates, especially in displaced ZMC fractures involving the orbital floor [41].

### Intraoperative imaging

The use of intraoperative 3D C-arm imaging proved beneficial, as demonstrated by the detection of inadequate reduction in six cases (three in each fixation group), prompting immediate revision. The benefit of intraoperative 3D imaging, as utilized as a standard procedure in our study, is increasingly supported by the literature [42]. The systematic review by Dubron et al. affirmed the utility of intraoperative navigation and imaging in identifying malreductions and improving anatomical outcomes, although cost and accessibility remain barriers [3]. The authors highlight the value of intraoperative imaging for real-time surgical quality control to enhance precision and reduce the need for secondary corrections.

In addition to clinical and radiological data, biomechanical modeling using finite element analysis (FEA) represents a valuable adjunct to minimally invasive surgical planning. When combined with intraoperative imaging, FEA enables a more precise assessment of fracture reduction by identifying optimal fixation points based on patient-specific fracture geometry and load distribution [4]. As demonstrated by Refahee et al., this integrative approach can enhance the accuracy and effectiveness of surgical interventions. Increasing adoption of digitally assisted and AI-integrated workflows raises the prospect of truly personalized trauma surgery. In contrast to conventional 2D accuracy analyses, as applied in our study, de Kort et al. introduced a novel method for three-dimensional symmetry assessment in zygomaticomaxillary complex (ZMC) fractures [10]. This approach may serve as an objective outcome parameter in future research and holds potential for real-time intraoperative guidance and correction.

### Strengths and limitations

Among the primary strengths of our study is the use of intraoperative 3D imaging, allowing real-time, objective 3D volumetric analysis of anatomical reduction. This approach minimizes reliance on external soft-tissue landmarks, which are subject to distortion from swelling, and allows quantification of hard-tissue alignment before wound closure. Furthermore, the comparative design within a single institution, with all procedures performed under standardized protocols, reduces procedural variability. The inclusion of volumetric and geometric parameters provides a comprehensive view of fixation outcomes.

This study provided critical insights into the evaluation of the institutional learning curve. We have been using the transoral (Keen’s approach) for ZMC fractures since 2007, and a supplementary analysis of outcomes stratified by year could highlight the impact of our internal learning curve. However, two constraints prevented us from conducting this analysis. Firstly, only data dating back to March 2019, when the department acquired a modern intraoperative C-arm, could be analyzed. Before that, the quality of intraoperative imaging was not sufficient for proper analysis. Secondly, the number of surgeries performed by each surgeon as well as the level of experience of each surgeon over time, varies significantly, which could lead to observation bias.

Our method of assessing zygomatic symmetry has been used in previous studies, was feasible in our clinical setting, and allowed reproducibility across our dataset, although we acknowledge that 3D volumetric analysis would be superior. Importantly, as a teaching institution, our residents are trained to apply conventional, well-documented measurement protocols, particularly when advanced 3D resources are not available. This reflects both the educational mission of our centre and the reality that such resource limitations are still common in clinical practice [28, 29].

Nonetheless, several limitations must be acknowledged. First, its retrospective nature inherently introduces selection bias, especially as the choice between 1-point and multiple-point fixation was not standardized but left to the operating surgeon’s intraoperative judgment. Although intraoperative 3D imaging offered objective assessment of reduction, the choice of fixation strategy and surgical outcomes were likely influenced by operator experience, as more than half of the cases were performed by residents, which may limit reproducibility despite imaging-based quality control. Second, a major limitation of this study is that only 2-dimensional linear measurements were performed; 3-dimensional volumetric analysis, which may offer a more accurate assessment of zygomatic bone asymmetry, was not technically feasible. The reliance on 2D linear measurements using reference lines that may be affected by individual anatomical asymmetry. Although this method has been used in prior studies and applied consistently here, it cannot achieve true 3D precision, and intra-/inter-observer reliability remains limited. Third, the relatively small sample size in the multiple-fixation group (*n* = 20) limits the statistical power for subgroup analyses. Additionally, no separate statistical analysis was performed for the small subgroups of Zingg Type A fractures (A2 and A3), which may restrict the generalizability of our findings to these specific fracture patterns. Fourth, while intraoperative data offer valuable insight into immediate anatomical accuracy, they do not account for long-term bone remodeling, functional outcomes, or patient satisfaction—parameters critical for comprehensive evaluation. Thus, the absence of long-term functional or patient-reported outcomes limit conclusions about the sustained aesthetic and functional benefit of different fixation strategies. Prospective research with 1-year follow-up assessment is needed to correlate the repositioning results with long-term clinical and functional outcomes. Fifth, we did not include a direct comparison group from other institutions that use a similar transoral approach. This limits the generalizability and benchmarking of our findings against external standards, and future multicenter studies could provide more robust comparative insights. Sixth, the measurements of the zygomatic bone as well as the registration and segmentation for the calculation of the intraorbital volume was conducted manually by one investigator based on his anatomical judgment. No artificial intelligence or fully automated software was used for this process. While this approach reflects practical clinical workflows and allows case-specific adjustments, it may introduce observer-dependent variability and subjective bias. Intraoperative imaging quality and semiautomated segmentation still rely on user input, introducing inter-operator differences. No additional analysis of intraobserver reliability was performed, which may limit the generalizability of our findings.

### Prospectives

Our data show that multiple-point fixation provides superior anatomical alignment, particularly in malar height and transverse symmetry, but this did not translate into higher revision rates or complications. No patients required secondary intervention, indicating that both fixation strategies can yield satisfactory functional and aesthetic outcomes when supported by intraoperative quality control. This supports a treatment concept oriented toward surgical minimalism.

In our institutional protocol, ZMC fractures are initially approached through a transoral approach with intraoral reduction and osteosynthesis at the zygomaticomaxillary buttress. This approach allows for an initial low-morbidity fixation. Intraoperative 3D imaging is routinely used to verify reduction and facial symmetry. Only if malreduction or rotational error is detected are additional fixation points added. This “escalation-based” strategy minimizes unnecessary exposure, reduces operative time, lowers morbidity, and limits scarring, aligning with current trends toward individualized, resource-efficient care guided by intraoperative feedback.

Future prospective studies with standardized protocols incorporating guided reduction and 3D planned patient-specific osteosynthesis, validated aesthetic scoring systems, patient satisfaction surveys and long-term follow-up are necessary to establish optimal fixation protocols tailored to fracture severity. Future research should also incorporate 3-dimensional volumetric analyses to allow a more precise evaluation of the intraoperative zygomatic bone symmetry and to validate the findings of this study. Advances in AI-assisted planning and intraoperative feedback systems hold promise for further optimizing outcomes in complex midfacial trauma.

## Conclusion

This study underscores the clinical importance of fixation strategy, surgical timing, and intraoperative 3D imaging in ZMC fracture management. Our results confirm that multiple-point fixation offers superior intraoperative accuracy in restoring malar geometry, particularly in complex fractures, compared with single-point fixation. Although both techniques can restore intraorbital volume, correction of zygomatic prominence—and thus facial symmetry—is more reliably achieved with multiple fixation points.

Single-point fixation at the zygomaticomaxillary buttress, however, remains a less invasive alternative for non-comminuted, minimally displaced fractures, especially when guided by intraoperative 3D imaging. The absence of secondary revisions in both groups highlights the adequacy of tailored, minimally invasive strategies when supported by real-time imaging and stepwise escalation.

Overall, these findings support a shift from rigid fixation protocols to adaptive, image-guided decision-making. Future studies should prospectively validate personalized treatment algorithms that integrate intraoperative imaging, patient-specific modeling, and AI-driven tools to optimize long-term outcomes while preserving resources and minimizing morbidity.

## Data Availability

The datasets used and/or analysed during the current study are available from the corresponding author on reasonable request.
